# Variants of the guanine riboswitch class exhibit altered ligand specificities for xanthine, guanine, or 2′-deoxyguanosine

**DOI:** 10.1073/pnas.2120246119

**Published:** 2022-05-27

**Authors:** Siddhartha Hamal Dhakal, Shanker S. S. Panchapakesan, Paul Slattery, Adam Roth, Ronald R. Breaker

**Affiliations:** ^a^Department of Molecular, Cellular and Developmental Biology, Yale University, New Haven, CT 06520-8103;; ^b^HHMI, Yale University, New Haven, CT 06520-8103;; ^c^Department of Molecular Biophysics and Biochemistry, Yale University, New Haven, CT 06520-8103

**Keywords:** aptamer, gene regulation, purine, transcription termination

## Abstract

The aptamer portions of previously reported riboswitch classes that sense guanine, adenine, or 2′-deoxyguanosine are formed by a highly similar three-stem junction with distinct nucleotide sequences in the regions joining the stems. The nucleotides in these joining regions form the major features of the selective ligand-binding pocket for each aptamer. Previously, we reported the existence of additional, rare variants of the predominant guanine-sensing riboswitch class that carry nucleotide differences in the ligand-binding pocket, suggesting that these RNAs have further diversified their structures and functions. Herein, we report the discovery and analysis of three naturally occurring variants of guanine riboswitches that are narrowly distributed across Firmicutes. These RNAs were identified using comparative sequence analysis methods, which also revealed that some of the gene associations for these variants are atypical for guanine riboswitches or their previously known natural variants. Binding assays demonstrate that the newfound variant riboswitch representatives recognize xanthine, guanine, or 2′-deoxyguanosine, with the guanine class exhibiting greater discrimination against related purines than the more common guanine riboswitch class reported previously. These three additional variant classes, together with the four previously discovered riboswitch classes that employ the same three-stem junction architecture, reveal how a simple structural framework can be diversified to expand the range of purine-based ligands sensed by RNA.

## Significance

Numerous purines and their metabolic derivatives must be monitored for proper control of relevant metabolic pathways. In certain bacteria, some metabolic steps related to purine production or degradation are catalyzed by proteins whose production is under direct regulatory control by purine-sensing riboswitches. Four riboswitch classes selective for guanine, adenine, and 2′-deoxyguanosine (two classes) reported previously exploit a common architecture involving a three-stem junction. Here, we describe three additional classes based on this same scaffold that sense xanthine, guanine, or 2′-deoxyguanosine. Thus, some riboswitches can diversify their ligand-sensing and gene-control functions without the need to evolve entirely novel structures, which highlights a capability that could have also been exploited by ancient forms of life during the RNA World.

Riboswitches are structured noncoding RNA domains most commonly found in the 5′-untranslated regions (UTRs) of mRNAs in bacteria and archaea (1–3). Each of these RNA devices typically carries a highly conserved aptamer domain that selectively binds a target ligand. In most instances, the aptamer partially overlaps an expression platform whose structure regulates the expression of an adjacent protein-coding gene in response to changing ligand concentrations (4–6). Unlike protein-based sensors, which have a diverse set of 20 amino acids to exploit when forming ligand-binding pockets, the known natural riboswitch aptamers (7) only rely on the four common nucleotides to form structures that selectively sense their targets. Because riboswitches are typically short-lived, they are unlikely to carry modified nucleotides, which are commonly present in tRNAs and rRNAs. The absence of modified nucleotides in riboswitches therefore places limitations on the diversity of ligand-binding pockets that can be formed by these RNA-based receptors.

Even with these restrictions, metabolite-binding riboswitches demonstrate a remarkable ability to recognize a wide range of biological ligands, such as signaling molecules, elemental ions, coenzymes, amino acids, and nucleotide derivatives (7). To date, more than 50 different riboswitch classes have been experimentally validated and more than 100 additional “orphan” (8) riboswitch candidates have been reported (9–11). Furthermore, it has been proposed that many hundreds of riboswitch classes have yet to be discovered just among the bacteria whose genomes have already been sequenced (7, 12, 13). Details on riboswitch classification guidelines used herein are provided in *SI Appendix*.

Presumably, these additional riboswitch classes will continue the currently observed trend that most ligands are related to ancient metabolites, which would have also been important in the RNA World (14, 15), a time before the evolutionary emergence of proteins. For example, recently reported riboswitch classes include those that sense the ubiquitous enzyme cofactors tetrahydrofolate (16) and nicotinamide adenine dinucleotide (NAD^+^) (17, 18), the thiamine pyrophosphate precursor HMP-PP (19), nucleoside diphosphates such as ADP (20), the purine degradation products xanthine and uric acid (21), and possibly the ribose derivative phosphoribosylamine (PRA) (22). Such RNA-like compounds were probably present in advanced representatives of RNA World organisms, and therefore perhaps these modern riboswitch aptamers are descendants from molecular sensors that existed long before proteins began to predominate as functional polymers (12, 23, 24).

The need to form precise binding pockets for cognate ligands that rarely change through evolution makes riboswitch aptamers among the most highly conserved biopolymer sequences and structures (12). Thus, when mutations occur at otherwise highly conserved nucleotide positions within the binding pockets of aptamers, it is likely that the variant RNAs have adapted to change their ligand-binding specificity (25). Examples of such evolutionary changes have been reported for several riboswitch classes, including guanine riboswitch variants that recognize adenine (26) or 2′-deoxyguanosine (25, 27), adenosylcobalamin riboswitch variants that sense aquocobalamin (28, 29), FMN riboswitch variants that sense chemical derivatives of this enzyme cofactor (25, 30–32), c-di-GMP riboswitch variants that sense c-AMP-GMP (33, 34), and guanidine-I riboswitch variants that sense ppGpp (35, 36), PRPP (36, 37), or ADP (20). Numerous additional changes in ligand specificities for variant riboswitch aptamers have also been proposed (17, 20, 25). These observations suggest that aptamer specificity changes are relatively common occurrences in evolution, whereby cells adapt preexisting riboswitch architectures to sense different ligands.

The apparent enormous potential for riboswitch aptamer variation and ligand specificity changes helps rationalize two somewhat contentious hypotheses. First, the relative ease with which evolution diversifies aptamer-binding pockets helps support the view that RNA World organisms could have evolved to become sophisticated entities without the help of protein factors to carry out ligand-sensing tasks (24). Second, fascile diversification of aptamer specificities makes it far more likely that bacterial species indeed carry hundreds or even thousands of novel riboswitch classes that remain to be discovered (12). These views are further supported by RNA engineers who have exploited riboswitch aptamer scaffolds to generate variant RNAs with altered ligand specificities (38, 39) or with novel functions (40).

Computational methods that identify conserved RNA sequence and structural features have been used to uncover the majority of riboswitch classes reported to date (e.g., refs. 41–43). However, it is challenging to identify variant riboswitch classes for several reasons. For example, it is often difficult to distinguish rare riboswitch variants when they are grouped with an abundant collection of representatives of another riboswitch class. Nevertheless, variant classes have been uncovered using a bioinformatics pipeline guided by atomic-resolution structures and unique gene associations (25). This method employs a search strategy wherein nucleotide positions located near the bound ligand in an atomic-resolution model for a known riboswitch aptamer class are examined for mutations. Presumably, these mutations in the ligand-binding pocket of the aptamer are indicative of a ligand-specificity change, which can be further supported if there is a corresponding change in the genes associated with these mutants or in the directionality of the genetic switch.

We previously reported the existence of several riboswitch variants that possibly represent novel riboswitch classes (25). One of these candidates is a guanine riboswitch (44) variant that has been proven to preferentially bind 2′-deoxyguanosine (25, 45), and this class has been named 2′-dG-II riboswitches. Herein, we provide evidence for the existence of three additional guanine riboswitch variant classes. These include variants that selectively sense: 1) xanthine, 2) guanine, and 3) 2′-deoxyguanosine. Although the variant class that senses guanine has not altered its primary ligand compared to the predominant class, the variant exhibits improved ligand discrimination. In addition, the novel 2′-deoxyguanosine variant binds guanosine with similar affinity, suggesting this RNA might sense the G nucleoside pool. These findings reinforce the hypothesis that some riboswitch aptamer architectures can readily adjust their ligand specificities by accruing mutations at key positions, enabling cells to more easily adapt by broadening their metabolite sensing and gene control capabilities.

## Results and Discussion

### Identification of Guanine Riboswitch Variants that Are Distinct from Known Classes.

**Fig. 1.**
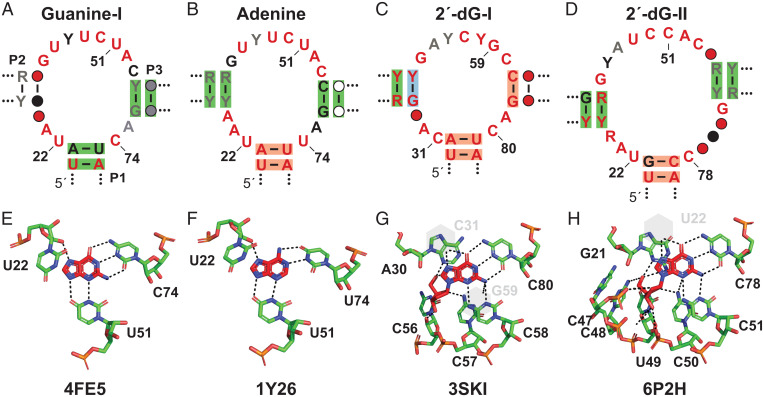
Consensus models and ligand-binding sites of guanine riboswitches or their variant classes that bind purine derivatives. (*A*–*D*) Consensus sequence and structural models for guanine (25), adenine (7), 2′-dG-I (27), and 2′-dG-II (25) riboswitches, respectively. Nucleotides depicted in red, black, and gray denote identities present in at least 97%, 90%, or 75% of the known representatives. Red, black, gray, or open circles indicate that nucleotides are present in at least 97%, 90%, 75%, or 50% of the representatives, respectively. Green shading indicates strong evidence of covariation consistent with conserved base-pairing. Blue shading indicates mutations occur that retain base-pairing, but that do not covary (e.g., a G-C to G-U change). Red shading identifies positions that likely base pair, but that do not experience mutations to reveal covariation. Some key nucleotide positions are annotated according to the numbering system used for their published structural models. (*E*–*H*) Atomic-resolution structural models for the guanine (46), adenine (47), 2′-dG-I (49), and 2′-dG-II (45) riboswitches, respectively. Protein Data Bank entry identifiers are provided for each structure. Guanine was modeled into the binding pocket determined for an RNA–ligand complex containing hypoxanthine (46).

Riboswitches that selectively sense guanine comprise one of the first classes of metabolite-responsive RNAs to be reported (Fig. 1*A*) (44). Guanine riboswitches are relatively abundant (7) and commonly control fundamental genes involved in purine biosynthesis, salvage, or uptake by reducing gene expression when guanine is plentiful in cells. Each uses a three-stem junction to position highly conserved nucleotides and form a binding pocket for this common purine molecule (44, 46, 47). Variant RNAs have also been discovered that conform to the general architecture of guanine riboswitches but that carry mutations in otherwise strictly conserved locations of the ligand-binding core. These variants function as riboswitches with altered ligand specificity, wherein one class senses adenine (Fig. 1*B*) (26, 47) and two classes sense the nucleoside 2′-deoxyguanosine, named 2′-dG-I (Fig. 1*C*) (27, 48, 49) and 2′-dG-II (Fig. 1*D*) (25, 45). Atomic-resolution structural models exist for all four classes (Fig. 1 *E*–*H*), which reveal the precise molecular recognition determinants for each ligand-binding site. The existence of such riboswitch variants provides support from natural evolution for the hypothesis that the common three-stem junction architecture used by these riboswitches serves as a robust and versatile scaffold for the formation of RNA aptamers (39).

**Fig. 2.**
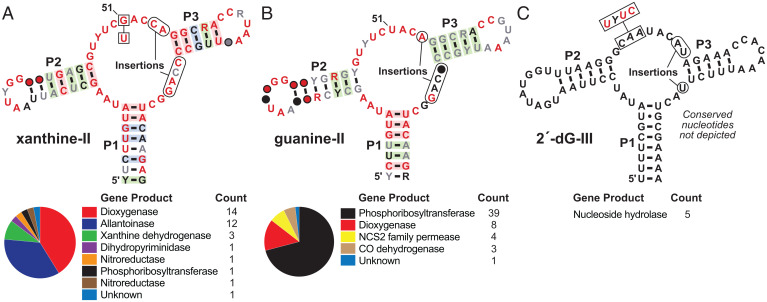
Consensus models and gene associations for three guanine riboswitch variant collections. (*A*, *Upper*) Consensus sequence and structural model for a variant riboswitch collection that selectively recognizes xanthine. A key nucleotide identity change (box) relative to guanine riboswitches is indicated, and two insertions are indicated. Additional annotations are as described for Fig. 1. (*Lower*) Gene associations for 34 representatives of the xanthine-II variant class depicted. (*B* and *C*) Information as described in *A* for variants that selectively bind guanine and guanosine, respectively. Gene associations were derived from 55 guanine-II and 5 guanosine aptamers. Genes listed are those that reside immediately downstream of the element, and exclude subsequent genes that might be present in operons. Note that the xanthine-II variant was originally reported as the “U, G, C” variant reported previously (25), whose function remained undetermined.

To search for novel guanine riboswitch variants, we used a comparative sequence analysis strategy (9). Specifically, we searched a genomic sequence database (RefSeq 96) to comprehensively identify RNA sequences that generally conform to the consensus guanine riboswitch sequence and structural model. By carefully inspecting thousands of possible aptamer representatives, three groups of unusual candidates were identified in the 5′-UTRs of genes from several *Paenibacillus* bacterial species as well as from *Propionispira* and unnamed *Clostridium* species (Fig. 2, *Upper*). Some notable sequence differences reside at positions known to directly interact with the ligand in guanine riboswitches reported previously. In addition, the genes associated with some of these variant RNAs are not typical of those regulated by guanine riboswitches, suggesting that the variants might have altered their ligand specificity (Fig. 2, *Lower*).

### Guanine Riboswitch Variants that Sense Xanthine.

One subset of the variant RNA representatives uncovered (25) (Fig. 2 *A*, *Lower*) frequently associates with genes annotated as coding for dioxygenase, allantoinase, and xanthine dehydrogenase enzymes, which are not commonly associated with guanine riboswitches. This variant RNA collection is also distinct because they carry nucleotide changes in the junction linking stems P2 with P3 (J2-3) and the junction linking stems P3 and P1 (J3-1) (Fig. 2*A*). For example, the nucleotide corresponding to position 51 of guanine riboswitches has changed from a U to a G (U51G). This nucleotide is known to directly form hydrogen bonds to the N3 and N9 positions of the purine ligand of guanine riboswitches (46, 47). In addition, there are substantial insertions in J2-3 and J3-1. Particularly noteworthy is the fact that guanine riboswitches and the previously reported variants that sense adenine or 2′-deoxyguanosine exploit a Watson–Crick base pair between a nucleotide in J3-1 and the ligand (26, 46, 47). The J3-1 insertion potentially disrupts this major ligand recognition determinant of guanine riboswitches. These mutations along with distinct downstream gene associations strongly suggested that the RNAs have undergone a change in ligand specificity.

**Fig. 3.**
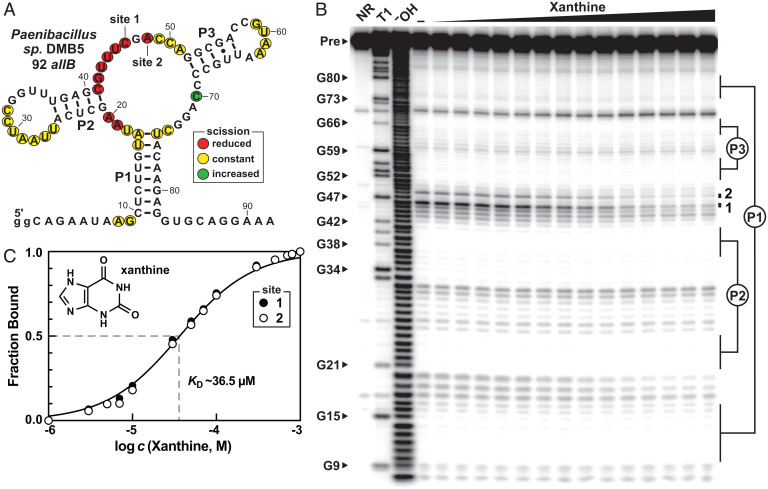
Selective binding of xanthine by a subset of guanine riboswitch variants. (*A*) Sequence and secondary structure model of the 92 *allB* RNA construct. To facilitate efficient in vitro transcription, guanine nucleotides (gg) were included at the 5′ terminus. The results of in-line probing assays mapped on the model were derived from the data presented in *B*. (*B*) 5′ ^32^P-labeled 92 *allB* RNAs were subjected to in-line probing reactions and analyzed using denaturing (8 M urea) PAGE in the absence (−), or in the presense of a range (1 µM to 1 mM) of xanthine concentrations. Lanes NR, T1, and ^‒^OH indicate no reaction, partial digestion with RNase T1 (which cleaves after G residues), and incomplete digestion under alkaline conditions (which cleaves after every nucleotide), respectively. The RNA precursor (Pre) band and bands corresponding to RNase T1 digestion after certain G nucleotides are indicated by arrowheads. Band intensity changes at sites 1 and 2 were quantified and used to estimate the fraction of RNAs bound to the ligand. (*C*) Plot of the normalized fraction of ligand bound to RNA versus the logarithm of the molar concentration of xanthine were used to estimate the *K*_D_ and stoichiometry of ligand binding. A theoretical binding curve with *K*_D_ of 36.5 µM representing one-to-one interaction (Hill coefficient of 1) is overlaid on the data points for comparison.

Often, the protein products of genes associated with riboswitch candidates provide clues regarding the identities of their ligands. Because the variants were found upstream of genes known to encode some enzymes associated with purine degradation (Fig. 2 *A*, *Lower*), we speculated that these variants could potentially recognize oxidized purine products. To test this hypothesis, we designed and prepared a 92-nucleotide RNA construct called 92 *allB* (Fig. 3*A*) that encompasses the putative riboswitch aptamer from the predicted *allB* gene (also called *pucH*) from *Paenibacillus* sp. DMB5. The RNA was subjected to in-line probing, which is a method that exploits the natural instability of RNA phosphodiester linkages to report on folding changes brought about by ligand binding (50, 51). Highly structured regions of RNA aptamers typically exhibit reduced rates of spontaneous RNA strand scission, and the analysis of band intensities upon cleavage product separation by PAGE can reveal evidence for ligand binding.

In-line probing reactions with 92 *allB* RNAs revealed that this aptamer becomes more structured in the presence of micromolar concentrations of xanthine (Fig. 3*B*). Specifically, the pattern of RNA cleavage product bands appears to be consistent with the proposed secondary structure model, wherein bands corresponding to strand scission after nucleotides in the J1-2 and J2-3 region undergo substantial reduction in intensity. Sites 1 and 2 (Fig. 3*A*), corresponding to cleavage after nucleotides 46 and 48, respectively, exhibited the greatest extents of band intensity changes. These sites were quantitated to estimate the fraction of RNA molecules bound to xanthine. This analysis indicates that the 92 *allB* aptamer exhibits a 1:1 binding curve with an apparent dissociation constant (*K*_D_) of ∼36.5 µM (Fig. 3*C*).

Importantly, the structure of 92 *allB* RNA modulates exclusively upon the addition of xanthine. The aptamer rejects guanine and other structurally similar purine analogs, each tested by in-line probing at a concentration of 100 µM (*SI Appendix*, Fig. S1). Presumably, the altered nucleotides in the joining regions of the three-stem junction form a novel and highly discriminatory binding pocket that efficiently excludes xanthine analogs. Given the considerable number of changes in the aptamer core and its strong discrimination against various purine analogs, we anticipate very little similarity between these other aptamer classes and the newly found xanthine aptamer. However, biophysical studies will likely be needed to reveal details regarding how the binding pocket differs from guanine riboswitches and the previously reported variant classes.

The existing bioinformatic and biochemical data, however, already strongly support the conclusion that the 92 *allB* construct is a representative of a second riboswitch aptamer class that senses xanthine. In some examples, we can identify potential terminator stems that partially overlap the aptamer, which suggests that these RNA aptamers function as the sensory components of riboswitches. The architectures of these RNAs are consistent with genetic “ON” switches, where ligand binding activates gene expression. Given the gene associations, it appears that these xanthine riboswitches participate in regulating the degradation or salvage of various purine compounds.

We also recently (21) reported the experimental validation of a riboswitch class previously called *NMT1* motif RNAs (9) that binds xanthine in addition to its close purine derivative uric acid. Although this established riboswitch class is associated with similar genes, its distinct sequence and structural characteristics, along with its broader ligand specificity, supports its placement into a different class. Indeed, the proposed atomic-resolution structural model for the xanthine riboswitch aptamer class reported previously is entirely different (52) from the three-stem junction architecture that appears to be retained by the xanthine-binding variants described above. Therefore, we recommend naming the previously reported xanthine riboswitch class as “xanthine-I,” and the guanine riboswitch variant described above as “xanthine-II,” to reflect the existence of two distinct classes for this ligand.

### Guanine Riboswitch Variants with Enhanced Guanine Selectivity.

We also uncovered another collection of variant RNAs that are remarkably similar to the xanthine-II riboswitch aptamers described above (Fig. 2*B*). However, this group differs from xanthine-II aptamers in two ways. First, the RNAs retain a U nucleotide at the position equivalent to nucleotide 51 of guanine riboswitches, but they still carry most other variations characteristic of xanthine-II RNAs. Notably, the variants retaining U51 only carry a single nucleotide insertion in J2-3, whereas xanthine-II riboswitches carry a two-nucleotide insertion in this region relative to guanine riboswitches. A second difference is that the variant RNAs retaining U51 have gene associations, most commonly those coding for phosphoribosyltransferase (PRT) enzymes (Fig. 2 *B*, *Lower*), that are more representative of guanine riboswitches (44) than of xanthine-I (21) and xanthine-II (Fig. 2 *A*, *Lower*) riboswitches.

**Fig. 4.**
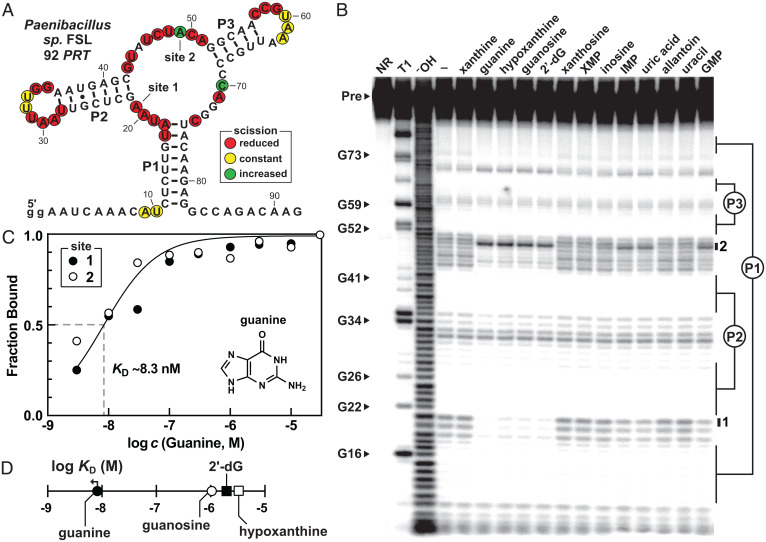
Variants carrying substantial sequence changes compared to the original guanine riboswitch consensus retain high-affinity guanine binding function. (*A*) Sequence and secondary structure model of the 92 *PRT* RNA construct. Other annotations are as described for Fig. 3. (*B*) In-line probing analysis of 5′ ^32^P-labeled 92 *PRT* RNA incubated in the absence (−), or in the presence of 100 µM of various compounds as indicated. (*C*) Plot of the normalized fraction of ligand bound to RNA versus the logarithm of guanine concentrations. The theoretical binding curve expected for 1:1 binding with an apparent *K*_D_ value of 8.3 nM is depicted. (*D*) Plot of the logarithm of the apparent *K*_D_ values for various ligands evaluated for binding by the 92 *PRT* RNA constuct. The arrow indicates that the actual *K*_D_ value is less than or equal to the than that indicated by the symbol.

The ligand-binding characteristics of a 92-nucleotide RNA construct carrying the aptamer variant from the *PRT* gene of *Paenibacillus* sp. FSL and called 92 *PRT* (Fig. 4*A*) were examined by in-line probing. Note that the annotation of this commonly associated gene indicates that the protein product likely functions as a PRT enzyme, which covalently attaches a 5-phosphoribose moiety to a nucleobase to produce a nucleotide. However, the nucleobase specificities for many annotated PRT enzymes, including the example discussed here, have not been established.

Initial in-line probing assays using 100-µM concentrations of candidate ligands revealed that xanthine is strongly rejected by the RNA, whereas guanine and several other purine analogs triggered modulation of band intensities in all three joining regions of the aptamer core (Fig. 4*B*). The *K*_D_ for guanine was estimated to be no poorer than ∼8.3 nM (Fig. 4*C* and *SI Appendix*, Fig. S2), although the full binding curve could not be established with the in-line probing reaction conditions used. This affinity compares favorably with that determined for a representative of the guanine riboswitch aptamer class originally reported (44). However, it is important to note that many riboswitches naturally operate as kinetically governed devices, and do not reach thermodynamic equilibrium (53, 54). Therefore, the *K*_D_ values reported here should be used for comparison purposes and are not necessarily indicative of the cellular concentrations of the ligands.

We also examined the binding characteristics of 92 *PRT* with various additional purine derivatives. Complete ligand-binding curves were generated for each of these guanine analogs, revealing that the compounds appear to bind with 1:1 stoichiometry (*SI Appendix*, Fig. S3). Furthermore, the analyses reveal that the *K*_D_ value for guanine is more than 100-fold better than the values determined for guanosine, 2′-deoxyguanosine, and hypoxanthine (Fig. 4*D*). Xanthine and numerous other purine derivatives do not show evidence of binding even at concentrations of 100 µM (Fig. 4*B*). Therefore, this unusual guanine-sensing aptamer discriminates against most other purine derivatives by at least 5 orders of magnitude. The previously described guanine binding pocket exhibits only ∼10-fold discrimination between guanine and the purine derivatives hypoxanthine and xanthine (44). These results strongly indicate that the members of this variant RNA group indeed function as guanine riboswitches. The variants appear to have adaptations compared to the previously reported guanine riboswitch class that permit them to retain guanine binding, but that also more effectively exclude similar purine compounds.

Given the unusual sequence and functional characteristics of the variant guanine aptamers, it might be reasonable to rename the previously reported guanine riboswitch class as “guanine-I” and name the variant riboswitches “guanine-II.” However, at this time, we cannot be certain that the two aptamer types indeed represent distinct classes, or if the variant RNAs make only modest changes to the ligand-binding site that would not merit a separate classification. For example, the banding pattern produced from in-line probing reactions is distinct in the J2-3 region for the guanine-II RNA construct examined herein (Fig. 4*B*) compared to the original guanine-I aptamer (44). This suggests that structural distinctions exist that merit different classification. However, the variant RNAs retain all the distinguishing sequence features in the regions linking the three stems as seen in guanine-I aptamers (Figs. 1*A* and 2*B*). This includes retention of the same nucleotide identities at positions contacting the guanine ligand, including U22, U51, and C74 (Fig. 1*E*). Thus, we use these riboswitch class names tentatively throughout the remainder of this report.

Notably, some species of *Paenibacillus* carry representatives of both guanine-I and guanine-II riboswitches (*SI Appendix*, Fig. S4). In these species, guanine-I riboswitches are associated with genes annotated as xanthine PRT (XPRTase). In contrast, guanine-II RNAs are found upstream of genes that encode PRT-type I family enzymes that include either hypoxanthine-guanine-xanthine PRT or xanthine-guanine PRT, among others. In addition, the directionalities of gene control upon ligand binding are predicted to be opposite for the guanine-I (OFF) and guanine-II (ON) riboswitches in *Paenibacillus* species.

It is currently not clear why some bacterial species use different guanine riboswitch variants to regulate PRT gene expression with opposite regulatory directions. One possibility is that abundant guanine triggers the guanine-II (ON) riboswitch to activate expression of a PRT gene whose product can directly utilize guanine for GMP formation (purine salvage pathway). In contrast, when guanine concentrations are too low, the guanine-I (OFF) riboswitch permits expression of a PRT gene whose XPRTase product converts other available purines such as xanthine into XMP, a metabolic intermediate that can subsequently be converted into GMP. Because it is more efficient to produce GMP by adding a 5-phosphoribose moiety to guanine compared to adding 5-phosphoribose to other nucleobases that subsequently need to be converted into GMP, guanine-sensing riboswitches with opposing regulatory directions likely enable cells to most efficiently produce GMP.

### A Variant of the Guanine Riboswitch that Senses 2′-Deoxyguanosine.

**Fig. 5.**
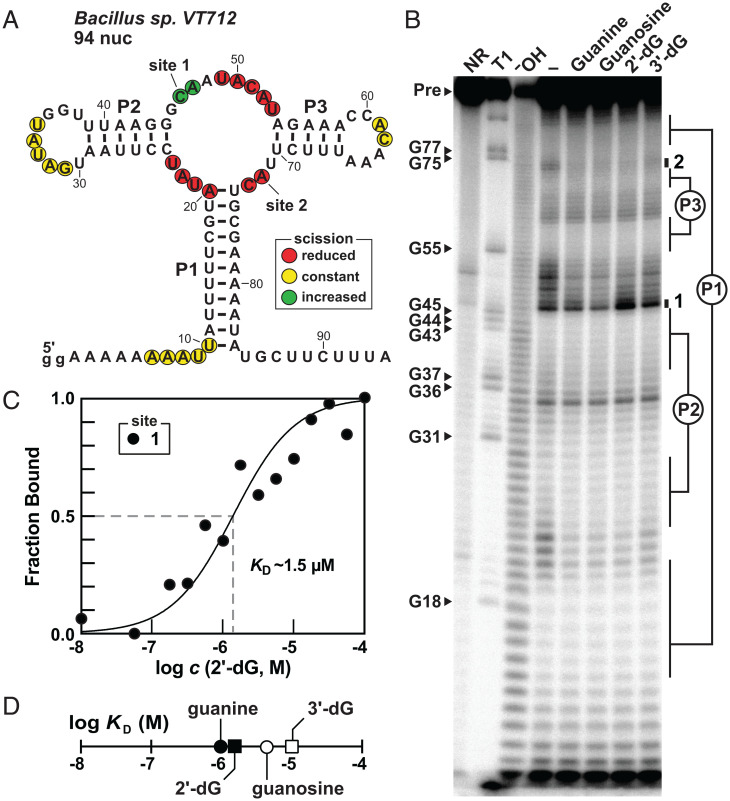
A rare variant exhibits improved affinity for guanosine and 2′-deoxyguanosine. (*A*) Sequence and secondary structure model of the 94 nuc RNA construct. Other annotations are as described for Fig. 3. (*B*) In-line probing analysis of 5′ ^32^P-labeled 94 nuc RNA incubated in the absence (−), or in the presence of 100 µM of various compounds as indicated. Note that the NR lane reveals a high background of RNA breakdown, which should be considered when interpreting the results of the in-line probing reactions containing candidate ligands. (*C*) Plot of the normalized fraction of ligand bound to RNA versus the logarithm of 2′-dG concentrations. The theoretical binding curve expected for 1:1 binding with an apparent *K*_D_ value of 1.5 μM is depicted. (*D*) Plot of the logarithm of the apparent *K*_D_ values for various ligands evaluated for binding by the 94 nuc RNA construct.

A third, rare cluster of riboswitch variants were identified that are exclusively associated with purine nucleoside hydrolase genes (Fig. 2*C*). These RNAs deviate from the sequence consensus of guanine riboswitches within all three joining regions. In-line probing analysis was conducted using an RNA construct called 94 nuc RNA derived from *Bacillus* sp*. VT712* (Fig. 5*A*). Again, the banding patterns observed from in-line probing assays are consistent with a three-stem junction. Based on this data and on the sequences present in the joining regions, we predict that the C nucleotide at position 73 of J3-1 likely forms a Watson–Crick base pair with the guanine moiety of the ligand, as is observed for several other classes based on this architecture (45–49).

In-line probing analyses also revealed that the RNA binds guanine more poorly than aptamers from either the guanine-I and guanine-II (Fig. 4*C*) classes, but exhibits improved affinities for guanosine, 2′-dG, and 3′-dG (Fig. 5*B*). Although guanine is bound with a slightly better *K*_D_ than 2′-dG, if the cellular concentration of 2′-dG is higher than that of guanine, then the riboswitch is likely to naturally respond to the nucleoside (or perhaps the pool of guanine-containing nucleosides, given their similar affinities), while ignoring free guanine, xanthine, and hypoxanthine.

Two observations support the conclusion that this variant class naturally responds to G-containing nucleosides. First, the gene exclusively associated with these RNAs codes for purine nucleoside hydrolase, which uses nucleosides as substrates and produces free nucleobases as products. Second, the aptamer region of these riboswitches precedes a predicted intrinsic terminator stem, wherein ligand binding is predicted to turn on expression of the downstream coding region (*SI Appendix*, Fig. S5). These observations indicate that the ligand is a nucleoside whose accumulation activates production of a nucleoside hydrolase. Thus, the genetic “ON” riboswitch likely detects excess guanine-containing nucleosides that need to be degraded, rather than activating a gene involved in producing more guanine when this compound is already in excess.

We speculate that the binding pocket for this variant class has distinct features compared to the binding pockets formed by 2′-dG-I (Fig. 1*G*) and 2′-dG-II (Fig. 1*H*). Specifically, the nucleotides within the J2-3 regions that form the binding pocket for the deoxyribose moiety and the exocyclic 2-amino and endocyclic N1 groups of the guanine ring are different among the three classes. This results in distinct ligand-binding specificity patterns for riboswitch aptamers known to respond to 2′-dG (*SI Appendix*, Fig. S6) Therefore, we recommend naming these additional variants 2′-dG-III riboswitches.

## Concluding Remarks

**Fig. 6.**
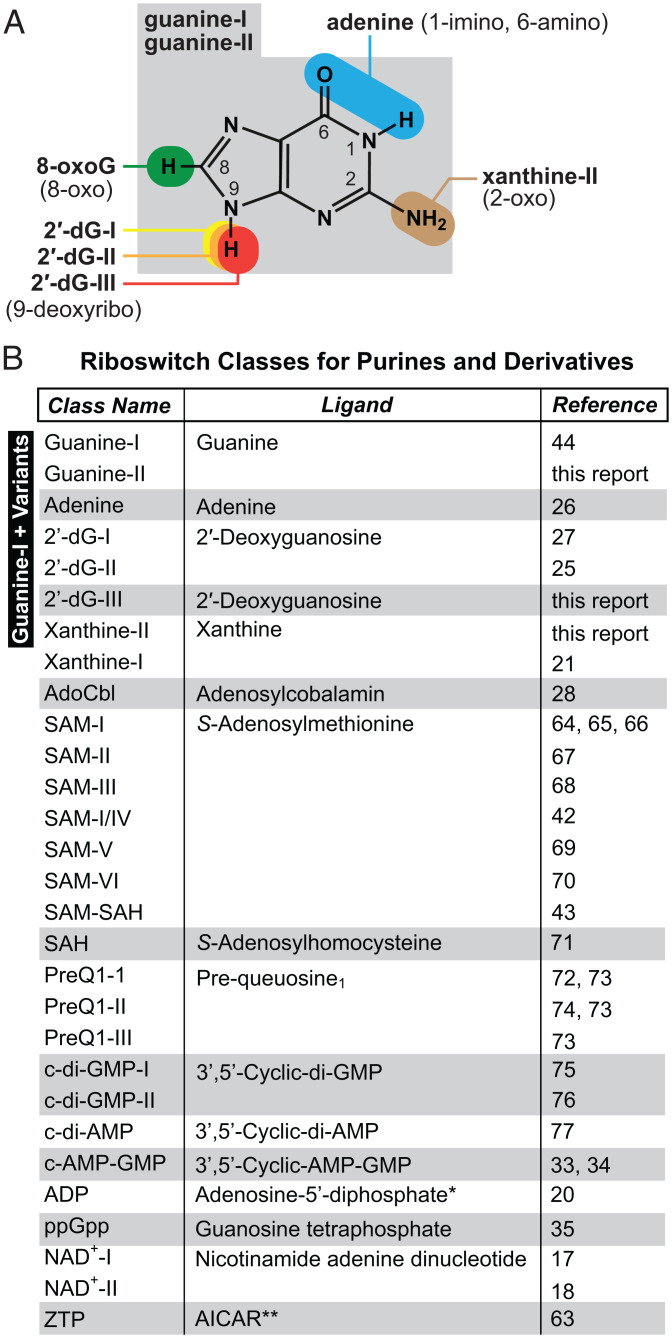
Recognition of purines and purine-related compounds by various riboswitch classes. (*A*) Chemical structure of guanine, the ligand for guanine-I and guanine-II riboswitches, and the sites of chemical differences for ligands recognized by riboswitch classes similar to the initial guanine-I riboswitch class. (*B*) Comprehensive list of riboswitch classes that sense purines, purine precursors, or purine-derived molecules (17, 18, 20, 21, 25–28, 32–35, 42–44, 62–76). *ADP riboswitch representatives examined can tightly bind adenosine-5′-diphosphate, 2′-deoxyadenosine-5′-diphosphate, and cytidine-5′-diphosphate (20). **ZTP riboswitches bind AICAR (aminoimidazole carboxamide ribonucleotide), also called ZMP (62), or its triphosphate form (63).

The identification and experimental validation of three additional aptamer types based on variants of the guanine-I riboswitch class adds to the tremendous diversity of riboswitches that are known to sense purines, purine precursors, or their derivatives (Fig. 6). Each of these newly identified RNAs appears to form the same architecture of a three-stem junction wherein the loops of P2 and P3 form a pseudoknot (kissing-loop structure) (46, 47) and the three joining regions largely form the ligand-binding pocket. Furthermore, we have also identified another variant class similar to guanine-I riboswitches that selectively senses the guanine oxidation derivative 8-oxoguanine. Thus, variant aptamers based on this simple three-stem junction form binding pockets that differently evaluate more than half the atomic centers that form the bicyclic structure of purines (Fig. 6*A*).

Although available crystal structures of the previously discovered variants (Fig. 1) provide some insights into the mechanisms by which the newly found purine variants might sense their natural ligands, it is difficult to accurately predict how the variant aptamers reported herein exploit the alterations in the joining regions to change their ligand-binding characteristics. Structural analyses will be required to reveal in detail how these variations reshape the ligand-binding pockets. There is also uncertainty regarding whether the variants were derived from a guanine-I riboswitch aptamer, or if they emerged independently. However, their occurrence exclusively in Firmicutes and their highly similar consensus sequence and structures suggests that the variants likely descend from a preexisting guanine-I riboswitch. Given the abundance of guanine-I riboswitches in many bacterial species, it seems much more likely that the variant riboswitches have emerged by accruing a few key mutations from this highly similar riboswitch class. Likewise, there could be many more examples of riboswitch variants that are currently hidden among the purine-sensing riboswitch sequence collections. Therefore, by implementing ever-improving computational search algorithms on the ever-increasing collections of genomic DNA sequences, it seems likely that additional rare variants will be discovered.

It is interesting to consider whether these RNAs emerged long ago, perhaps during the RNA World (14, 15) stage of life on Earth, or if they are more recent evolutionary adaptations of preexisting riboswitches. Given their narrow distribution in species of *Paenibacillus* and a few other organisms, these particular variant examples most likely have emerged more recently through the acquisition of mutations by guanine-I riboswitch aptamers. Regardless, this demonstrated potential for ligand-sensing diversification by natural RNA aptamers could have readily been exploited by RNA World organisms (24). Organisms whose metabolic pathways and processes were dominated by RNA and its close derivatives would have required the ability to sense many different purine or purine-like molecules to manage complex functions.

The remarkable diversity of riboswitch classes for purines and related compounds also could be due in part to the relative ease with which evolution can identify nucleic acid structures that bind to purines. It has been shown that directed evolution experiments more frequently yield nucleic acid binding pockets for purines than for pyrimidines (55, 56). Furthermore, purine-containing signaling molecules—such as c-di-GMP, c-di-AMP, c-AMP-GMP, ppGpp, and cAMP, which have been proposed to be ancient signaling molecules from the RNA World era (57, 58)—clearly predominate over signaling molecules incorporating pyrimidines. Riboswitch classes for four of the five purine-containing signaling molecules have been reported (Fig. 6*B*), suggesting that their emergence as important second messengers in today’s cells might be due in part to the ease of establishing RNA receptors for them early in evolution.

The apparent bias toward riboswitch ligands that carry purine moieties extends to enzyme cofactors, which are also extensively monitored by bacterial riboswitches (Fig. 6*B*). Given RNA’s limited chemical and structural diversity, it is not surprising that other riboswitch aptamer architectures also have diversified to selectively sense more than one purine-containing ligand. For example, variants of c-di-GMP-I riboswitches are known that sense c-AMP-GMP (33, 34), and riboswitches that sense ppGpp (35, 36) or ADP (20) are based on the same *ykkC* motif scaffold (41). Exploiting variants of a few aptamer scaffolds to diversify the types of purines that can be sensed would reduce the evolutionary burden required to produce novel aptamer architectures for each new molecular target. Again, a combination of preferential binding characteristics and the repurposing of existing aptamer scaffolds to sense close ligand derivatives might have allowed purines to become the preferred moieties associated with signaling molecules and enzyme cofactors, which persist in modern cells due to this evolutionary history.

## Materials and Methods

### Bioinformatics.

Automated homology search algorithms such as CMfinder (59) and Infernal (60) were used to identify sequences that are similar to guanine riboswitches from bacterial DNA sequence databases (RefSeq v80 and RefSeq v96), as previously described (9). The resulting hits were then manually scrutinized for unusual variants and for unusual gene associations. R2R software (61) was used to construct initial RNA consensus sequence and secondary structure models, which were examined and adjusted after comparison among all variants.

### Chemicals and Oligonucleotides.

All candidate ligands and synthetic oligonucleotides were purchased from Sigma-Aldrich. [γ-^32^P] ATP (specific activity: 6,000 Ci/mmol) was purchased from PerkinElmer.

### RNA Oligonucleotide Preparation.

To prepare RNA constructs, synthetic DNA templates base-paired to a T7 RNA polymerase promoter oligonucleotide were used in in vitro transcription reactions with T7 RNA polymerase following the protocol described previously (20). The resulting RNAs were purified by separation by denaturing 10% PAGE. The desired product bands were excised, the RNAs were eluted using crush-soak solution (200 mM NH_4_Cl, 10 mM Tris⋅HCl [pH 7.5 at ∼20 °C], 1 mM EDTA), and the RNAs were precipitated using ethanol. Next, 60 pmoles of the RNA transcripts were dephosphorylated using rAPid alkaline phosphatase (Roche Life Sciences) following the manufacturer’s protocol, and 5 pmoles of the resulting RNAs were 5′ ^32^P-radiolabeled using T4 polynucleotide kinase (New England Biolabs). Radiolabeled RNAs were purified using denaturing 10% PAGE gel, further prepared as described above, and resuspended in autoclaved dH_2_O for storage at −20 °C until use.

### In-Line Probing Assays.

Stock solutions of ligands were prepared by dissolving in 50 mM aqueous NaOH and then the desired ligand concentrations were made by serially diluting in 25 mM aqueous NaOH. Following the protocol described previously (50, 51), 5′ ^32^P-labeled RNAs were subjected to in-line probing reactions with the desired ligands in a 10-µL reaction mixture containing 100 mM Tris⋅HCl (pH 8.3 at 20 °C), 100 mM KCl, and 20 mM MgCl_2_ and incubated at room temperature for ∼40 h. Spontaneously cleaved RNA fragments were resolved using 10% PAGE and visualized using Typhoon FLA 9500 (GE Healthcare Life Sciences). ImageQuant 5.1 was used to quantify the cleavage patterns and GraphPad Prism 9 was used for graphical analyses.

## Supplementary Material

Supplementary File

## Data Availability

All experimental data are included in the main text and *SI Appendix*. RNA sequence alignments will be made available on request.
