# Supplementary material for: Variants of the guanine riboswitch class exhibit altered ligand specificities for xanthine, guanine, or 2′-deoxyguanosine
Source: Proc Natl Acad Sci U S A. 2022 May 27;119(22):e2120246119. doi: 10.1073/pnas.2120246119 (PMC9295807; doi:10.1073/pnas.2120246119)
Supplement: Supplementary File [file pnas.2120246119.sapp.pdf]

Supplemental Information

Variants of the guanine riboswitch class exhibit altered ligand specificities for xanthine, guanine or 2′-deoxyguanosine

Siddhartha Hamal Dhakal[a], Shanker S.S. Panchapakesan[a], Paul Slattery[a], Adam Roth[b], and Ronald R. Breaker[a,b,c,1]

[a]Department of Molecular, Cellular and Developmental Biology, Yale University, New Haven, CT 06520-8103, USA;
[b]Howard Hughes Medical Institute, Yale University, New Haven, CT 06520-8103, USA;
[c]Department of Molecular Biophysics and Biochemistry, Yale University, New Haven, CT 06520-8103, USA

[1]To whom correspondence may be addressed. Email: ronald.breaker@yale.edu

Riboswitch Classification

The classification and naming of riboswitches is inherently problematic and therefore we provide some details on how we address this challenge. There are several levels of consideration to be made when classifying riboswitches, and organizational decisions are sometimes made by research groups that have different opinions on classification. At the highest level of organization, there is disagreement on what constitutes a "riboswitch". The term was originally defined (1) to include only RNA switches that senses small molecules or ions without the action of a protein receptor. In subsequent years, some researchers have broadened the definition to include protein-responsive RNAs, attenuators, sRNA binding sites, thermosensors, and so on. We use the original definition of the "riboswitch" term when discussing the findings described in the current report.

Given this original riboswitch definition, we then organize riboswitches based on the structures and functions of their aptamer domains. This organizational level also poses challenges because there can be multiple different riboswitch aptamer structural "classes" that sense the same ligand, or similar structures for riboswitches that sense different ligands. We have addressed this challenge in part by naming each riboswitch after the ligand it binds. Therefore, similar-looking RNA structures that bind different ligands are considered different classes, and they carry the name of their distinct ligands. Also, different structural forms that bind the same ligand are likewise defined as different classes. These classes are designated by using Roman numerals after the ligand name, such as SAM-I, SAM-II, SAM-III, and so on (2-5). It is our opinion that this naming convention is most easily understood by scholars because it avoids the need to look up obscure riboswitch names when pursuing scholarship in this field. Specifically, the name of the riboswitch class carries information on the ligand that is sensed and the structural class to which it belongs.

Unfortunately, additional organizational complexity is caused because some riboswitch aptamers have structural features that look distinct, but that do not alter the ligand binding selectivity of the riboswitch. This might be in the form of an additional substructure, or a distinct nucleotide sequence in a region of the aptamer that is otherwise well conserved. Creating precise definitions for sequence and structural differences that merit organizational distinctions is not practical, but we have frequently used an empirical measure: If computational algorithms yield separate consensus 'clusters', then the differences might merit separate notations such as "class" or "type". To address this issue, we have used the following considerations. If these distinct sequence and structural features do not substantially alter the ligand binding pocket or the global

structure of the aptamer, they are considered to be different "types" of the same class (e.g. $PreQ_1$-I, types 2 and 3). Otherwise, they merit assignment into separate classes as described above.

However, sometimes different types were assigned to highly similar riboswitch candidates that subsequently proved to bind different ligands, and these RNAs were subsequently renamed as distinct classes. For example, the *ykkC* motif class of riboswitch candidates (6) was revealed to include several distinct "subtypes" as determined based on differences in consensus models and distinct gene associations (7). Each subtype has since been proven to bind (7-10) or is suspected of binding a different ligand, and therefore each merits a separate class assignment. In other words, the historical progression of riboswitch discovery and validation work further confounds the effort to provide a clear classification and nomenclature system for all riboswitches.

In the current report, we also use the term "variant" to refer to the fact that the RNAs under examination exhibit recognizable nucleotide sequence variations of the commonly found riboswitch class for guanine (11). This term is not meant to be an organizational level that is distinct from a "class" or a "type".

The considerations described above are already highly subjective and are made even more challenging by the fact that RNA secondary and tertiary structures commonly exhibit high adaptability through evolution. Specifically, the inherent structural versatility of RNA is sufficient to give rise to dozens of experimentally validated riboswitch classes (12). Therefore, RNA molecules can form diverse structures and functions that blur the lines between the organizational levels presented above. Although this causes frustration for scholars who seek to perfectly classify riboswitches, the structural versatility of RNA means that natural evolution, as well as synthetic biologists, can exploit this capability to form remarkably diverse types of riboswitches for many applications.

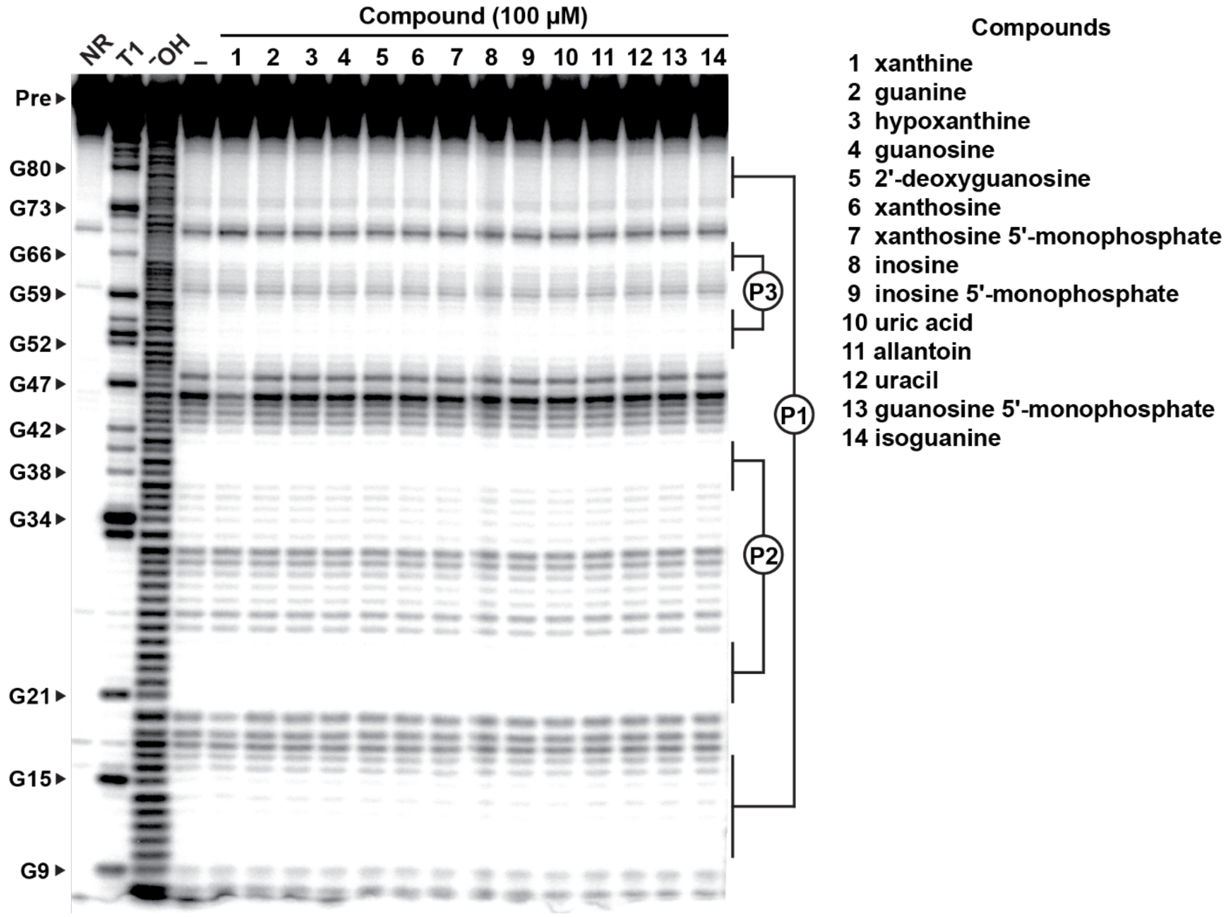

Fig. S1. Structure-activity relationship (SAR) analysis of the 92 *allB* xanthine riboswitch aptamer by using in-line probing assays. (Left) Analysis of products generated by in-line probing assays with 5′ ^{32}P- labeled RNAs resolved by denaturing PAGE using various compounds at 100 µM. Annotations are as described in **Fig. 3*B***. (Right) List of compounds examined using in-line probing analyses.

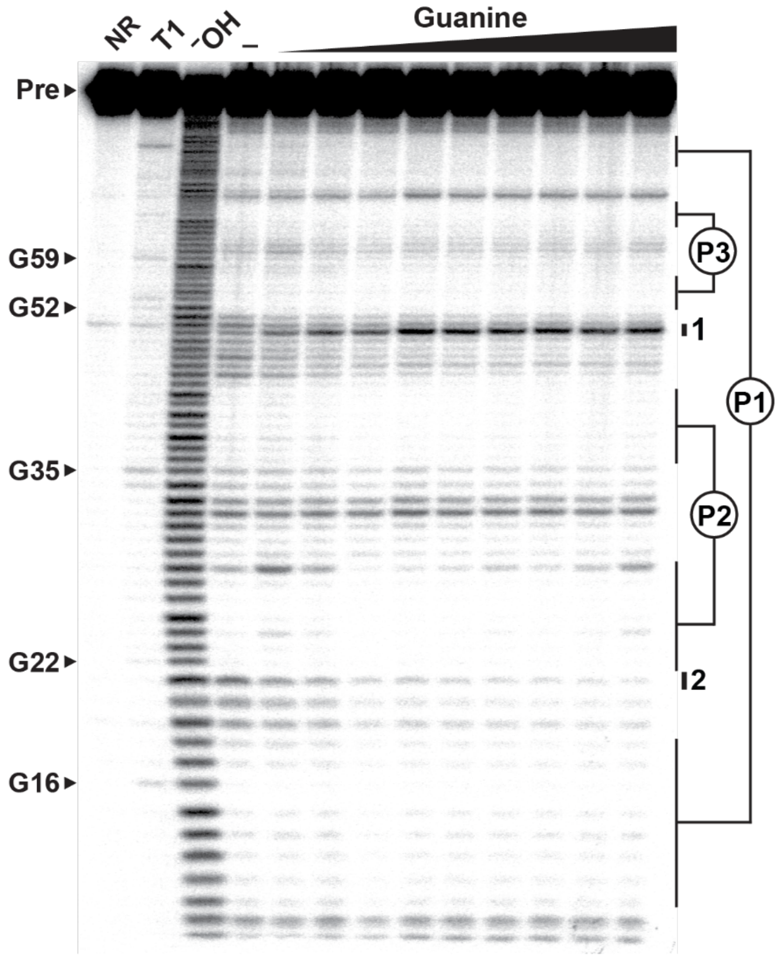

Fig. S2. Guanine binding analysis of 5′ ^{32}P- labeled 92 *PRT* RNA by in-line probing. Guanine concentrations ranged from 3 nM to 30 µM. **S**ites 1 and 2 used for estimating the fraction of RNA bound to ligand (**Fig. 3*C***) are indicated. Other details and annotations are as described for **Fig. 3*B*.**

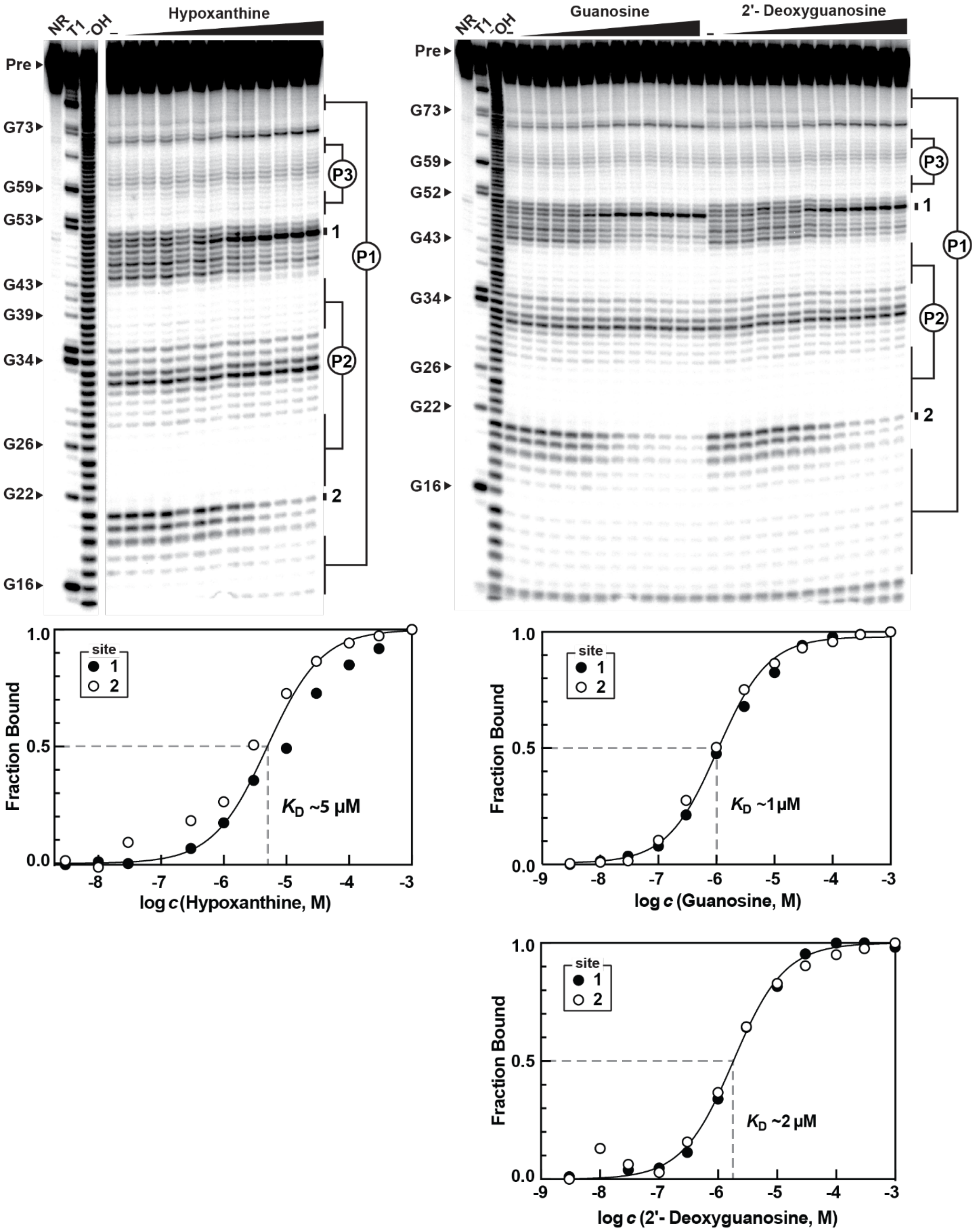

Fig. S3. (Legend on next page)

Fig. S3. In-line probing assays of radiolabeled 92 *PRT* RNA with several purine derivatives. Top: PAGE autoradiograms depicting the separation of the products of in-line probing reactions using 5′ ^{32}P-labeled 92 *PRT* RNA with various concentrations (3 nm to 1 mM) of hypoxanthine, guanosine, or 2′-deoxyguanosine as indicated. Annotations are as described for **Fig. 3*B***. Band intensities at sites 1 and 2 were used to estimate K_D values. Bottom: Plots of the estimated fraction of RNAs bound to ligand versus the logarithm of the concentration (*c*) of the indicated ligands.

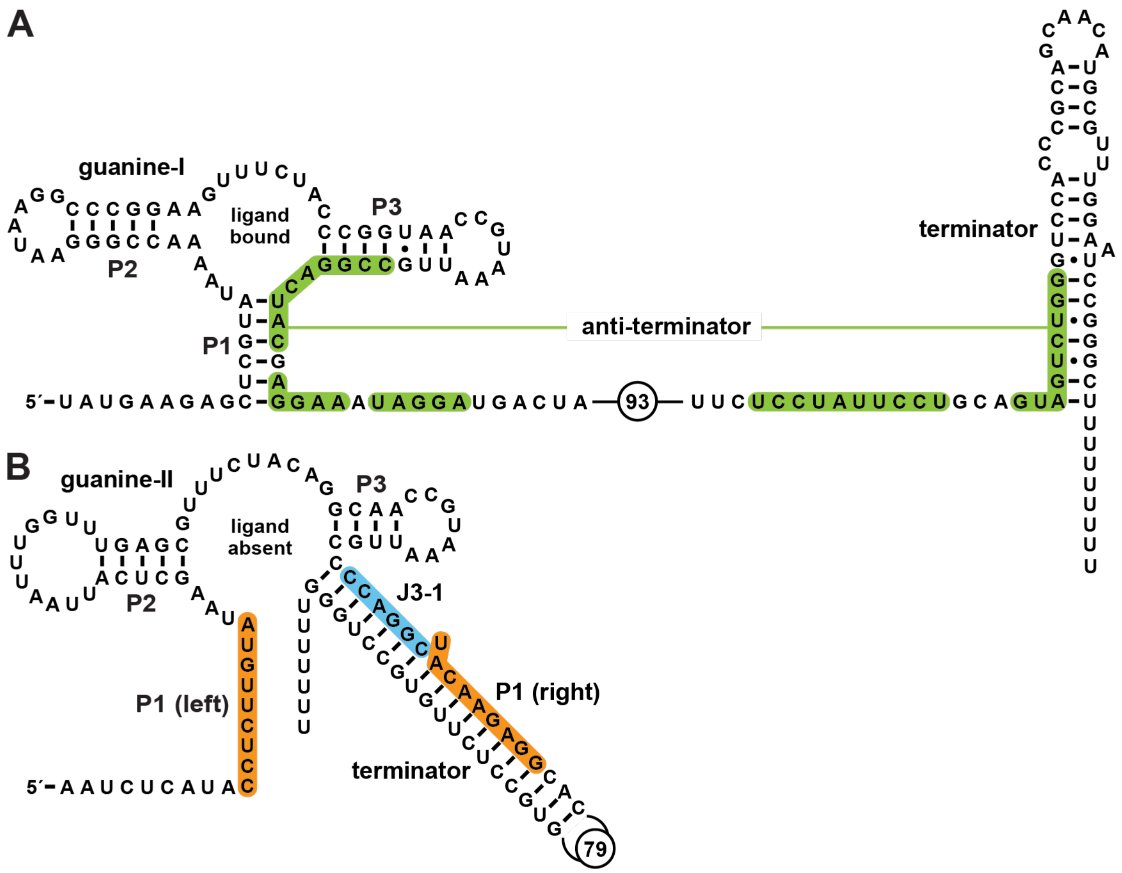

Fig. S4. Sequences, predicted secondary structure models, and proposed expression platforms for guanine-I and guanine-II riboswitches from *Paenibacillus Sp.* HW567. (*A*) Sequence and secondary structure of a representative guanine-I riboswitch class is consistent with a genetic "OFF" switch wherein ligand binding permits the formation of a strong terminator stem. The possible competing anti-terminator structure is depicted with green shading. (*B*) Sequence and secondary structure model of a guanine-II riboswitch is consistent with a genetic "ON" switch wherein ligand binding would preclude formation of the terminator stem. Specifically, the ligand-bound aptamer structure requires that the orange-shaded nucleotides form a complete P1 structure, and the blue-shaded nucleotides form J3-1.

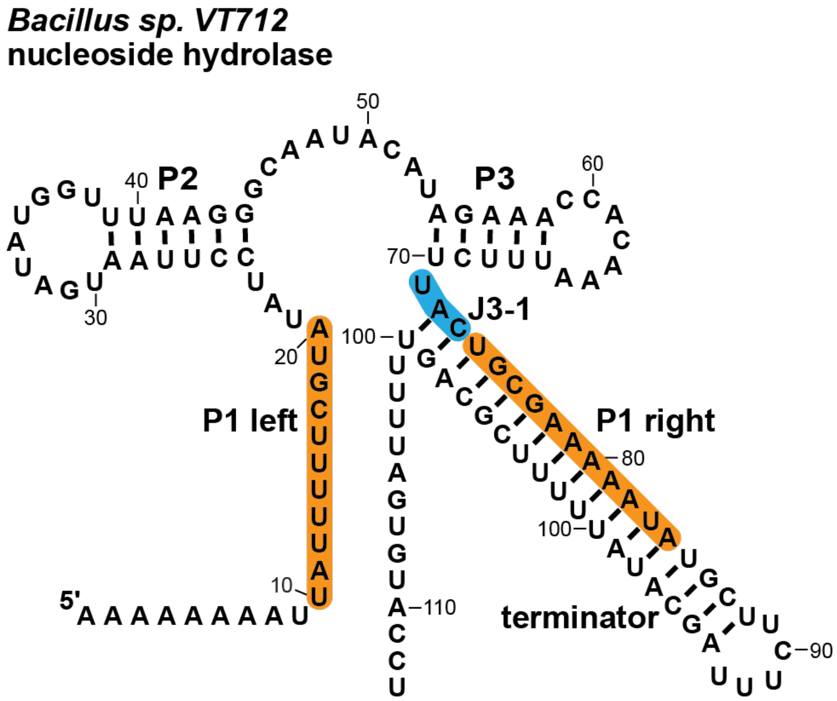

Fig. S5. Predicted mechanism for the 2′-dG-III riboswitch from the *Bacillus sp. VT712* nucleoside hydrolase gene. The predicted terminator stem forms at the expense of the P1 stem of the aptamer (orange shading), which is consistent with ligand binding turning "ON" gene expression. Other annotations are as described for ***SI Appendix,*** **Fig. S4**.

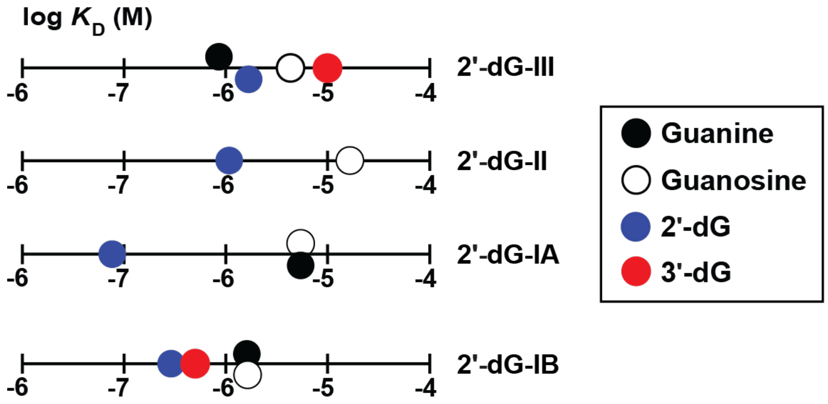

Fig. S6. Apparent dissociation constant (K_D) values for representatives of the three main classes of riboswitches that sense 2′-dG. Values for 2′-dG-III are derived from **Fig. 5*D***. Values for 2′-dG-I (13) and 2′-dG-II (14) were published previously.

SI References

1. A. Nahvi, N. Sudarsan, M. S. Ebert, X. Zou, K. L. Brown, R. R. Breaker, Genetic control by a metabolite binding mRNA. *Chem. Biol.* **9**, 1043-1049 (2002).
2. K. A. Corbino, et al., Evidence for a second class of *S*-adenosylmethionine riboswitches and other regulatory RNA motifs in alpha-proteobacteria. *Genome Biol.* **6**, R70 (2005).
3. E. Poiata, M. M. Meyer, T. D. Ames, R. R. Breaker, A variant riboswitch aptamer class for *S*-adenosylmethionine common in marine bacteria. *RNA* **15**, 2046-2056 (2009).
4. G. Mirihana Arachchilage, M. E. Sherlock, Z. Weinberg, R. R. Breaker, SAM-VI RNAs selectively bind *S*-adenosylmethionine and exhibit similarities to SAM-III riboswitches. *RNA Biol.* **15**, 371-378 (2018).
5. J. X. Wang, E. R. Lee, D. R. Morales, J. Lim, R. R. Breaker, Riboswitches that sense *S*-adenosylhomocysteine and activate genes involved in coenzyme recycling. *Mol. Cell* **29**, 691-702 (2008).
6. J. E. Barrick et al., New RNA motifs suggest an expanded scope for riboswitches in bacterial genetic control. *Proc. Natl. Acad. Sci. USA* **101**, 6421-6426 (2004).
7. J. W. Nelson, R. M. Atilho, M. E. Sherlock, R. B. Stockbridge, R. R. Breaker, Metabolism of free guanidine in bacteria is regulated by a widespread riboswitch class. *Mol. Cell* **65**, 220-230 (2017).
8. M. E. Sherlock, H. Sadeeshkumar, R. R. Breaker, Variant bacterial riboswitches associated with nucleotide hydrolase genes sense nucleoside diphosphates. *Biochemistry* **58**, 401-410 (2019).
9. M. E. Sherlock, N. Sudarsan, R. R. Breaker, Riboswitches for the alarmone ppGpp expand the collection of RNA-based signaling systems. *Proc. Natl. Acad. Sci.* USA **115**, 6052-6057 (2018).
10. M. E. Sherlock, N. Sudarsan, S. Stav, R. R. Breaker, Tandem riboswitches form a natural Boolean logic gate to control purine metabolism in bacteria. *eLife* **7**, e33908 (2018).
11. M. Mandal, B. Boese, J. E. Barrick, W. C. Winkler, R. R. Breaker, Riboswitches control fundamental biochemical pathways in *Bacillus subtilis* and other bacteria. *Cell* **113**, 577-586 (2003).
12. P. J. McCown, K. A. Corbino, S. Stav, M. E. Sherlock, R. R. Breaker, Riboswitch diversity and distribution. *RNA* **23**, 995-1011 (2017).
13. J. N. Kim, A. Roth, R. R. Breaker, Guanine riboswitch variants from *Mesoplasma florum* selectively recognize 2′-deoxyguanosine. *Proc. Natl. Acad. Sci. USA* **104**, 16092-16097 (2007).
14. Z. Weinberg, J. W. Nelson, C. E. Lunse, M. E. Sherlock, R. R. Breaker, Bioinformatic analysis of riboswitch structures uncovers variant classes with altered ligand specificity. *Proc. Natl. Acad. Sci. USA* **114**, E2077-E2085 (2017).
